# The Effect of Magnesium Sulfate on Renal Colic Pain Relief; a Randomized Clinical Trial 

**Published:** 2017-01-10

**Authors:** Abolfazl Jokar, Ali Cyrus, Maryam Babaei, Majid Taheri, Amir Almasi-Hashiani, Ezatollah Behzadinia, Arash Yazdanbakhsh

**Affiliations:** 1 Department of Emergency Medicine, Arak University of Medical Sciences, Arak, Iran.; 2 Medical Ethics and Law Research Center, Shahid Beheshti University of Medical Sciences, Tehran, Iran.; 3 Department of Epidemiology and Reproductive Health, Reproductive Epidemiology Research Center, Royan Institute for Reproductive Biomedicine, ACECR, Tehran, Iran.

**Keywords:** Renal colic, magnesium sulfate, therapeutics

## Abstract

**Introduction:**

Renal colic can be managed by preventing the contraction movements of ureter muscles. By reducing acetylcholine in the nerve terminals, magnesium sulfate could be effective in this regard. The aim of this study is to investigate the effect of magnesium sulfate on acute renal colic pain relief.

**Method:**

The present study was a double-blind clinical trial in which the patients suffering from acute renal colic were randomly divided into 2 groups of who either received standard protocol (intravenous infusion of 0.1 mg/Kg morphine sulfate, 30 mg of Ketorolac, and 100 ml normal saline as placebo/15 minutes) or standard protocol plus 15 mg/Kg of intravenous magnesium sulfate 50%/100 ml normal saline/15 minutes. Severity of patients’ pain was measured by visual analogue scale (VAS) at baseline, and 30 and 60 minutes after infusion. The collected data were analyzed using STATA statistical software.

**Results:**

100 cases were randomly allocated to intervention or control group. The two groups were similar in baseline pain score and demographic characteristics. At 30 and 60 minutes, mean pain score was less in the intervention group compared to the control group. Moreover, the difference between the two groups was statistically significant regarding the additional amount of morphine, suggesting that the intervention group needed less additional morphine than the control group.

**Conclusion:**

The results of this study showed that Magnesium sulfate can be used as an adjunct drug in treatment of patients suffering from renal colic. It not only alleviates the pain in the patients, but also diminishes the need for pain medications.

## Introduction

Urinary tract stone is a common urological disease, which is often symptomized through twinges localized to the sides or a radicular pain towards groins and genitalia, which is referred to as renal colic (1). Due to the obstruction of urine flow and thereby increased ureteral wall traction, renal colic is formed above the point of obstruction. This increased pressure surges the production and local release of prostaglandins, which brings about dilation of blood vessels and diuresis, resulting in a further increase in the pressure inside the kidney. High levels of prostaglandins also play a role in development of edema around the stones (2). In addition, long isotonic contractions above the point of obstruction increase the production of lactic acid, contributing to slow type A and fast type C nerve fibers’ stimulation and introduction of more pain (3). A wide variety of medications are available to treat the pain associated with acute renal colic, each of which affect different parts of the mechanisms causing the pain. For the time being, opioids and non-steroidal anti-inflammatories drugs (NSAIDs) are the main drugs in treatment of renal colic. Opioids are cheap and measurable drugs; nevertheless, they are addictive and may have side effects such as nausea, vomiting, constipation and drowsiness. In higher doses, they can even cause respiratory depression. Furthermore, opioids have no effect on the cause of pain while they may have contractile effects on the ureteral tone (4). NSAID, on the other hand, having a direct effect on prostaglandins release, can bring about pain relief through reducing renal pressure and diuresis. They may also reduce the edema of ureter around the stones (2). Despite all these advantages, however, these drugs may induce some secondary regulatory responses in the kidney leading to some obstructions (5). Considering that renal colic can be caused by peristalsis movements above the point of obstruction (1), it is hypothesized that it is possible to control the patients’ pain by preventing the contraction movements in the ureters. By the same token, tocolytic drugs such as magnesium sulfate can be effective in this regard. It prevents calcium from entering the smooth muscle cell membrane, activates adenylate cyclase and cyclic AMP, and increases the uptake of calcium by sarcoplasmic network (6). Moreover, reducing acetylcholine in the nerve terminals, magnesium sulfate can also decrease muscle contractions (5). Based on above-mentioned facts, the aim of the present study is to investigate the effect of magnesium sulfate on acute renal colic pain relief.

## Methods

***Study design and setting***

This randomized double-blind clinical trial was designed to investigate the effect of intravenous magnesium sulfate in pain relief of patients presenting to emergency department (ED) following renal colic. 

**Ethical Consideration**

Before being included in the study, all the patients were provided with proper explanation about the study and they signed the informed consent for being included. They were free to decline to participate and to withdraw from the study. All the research group members were required to comply with all the provisions of the Declaration of Helsinki and Arak University of Medical Sciences research ethics. In addition, this project has been approved by Arak University of Medical Sciences Ethics Committee (Code: Arakmu.ac.1394.44). This study is registered on Iranian registry of clinical trials with the registration number: IRCT2016020223552N6.

***Participants***

Patients suffering from acute renal colic admitted to the ED of Vali-Asr Hospital, Arak, Iran, comprised the target population of this study. The cases were enrolled using convenient sampling, considering some inclusion and exclusion criteria. 

Inclusion criteria were as follows: being clinically diagnosed as renal colic, being between 18 to 55 years old, and having a pain severity > 5 based on visual analogue scale (VAS) (7).

Exclusion criteria were as follows: a history of seizure; any heart, liver, kidney or metabolic disease; fever (oral temperature > 38 Celsius), systolic blood pressure less than 90 mm Hg, pregnancy, acute abdomen, paregoric drug consumption 3 hours before presenting to ED, history of addiction or drug allergy, and having taken calcium channel blockers.

Renal colic was clinically defined through diagnosis of such practical benchmarks as twinges localized to the sides or radicular pain towards groins, lower abdomen and testicles with probable associated symptoms like nausea, vomiting, sweating, pallor, dysuria, frequent urination, urgency of urination, and blood in the urine. Suspected cases of acute coronary syndrome were ruled out with electrocardiography.

**Randomization** **and Sequence generation**

Patients were randomly allocated to two groups using a balanced block randomization technique. To do that, they were divided into blocks of 6. Allocation of the subjects into two groups was done with the help of an online application called “Sealed Envelope” (8). In this study, as a result of using balanced block randomization and allocating unique codes to each individual, “allocation concealment” was carried out. Owing to random allocation, distribution of potential confounding variables is considered to be identical in the two groups and their confounding role is controlled.

**Implementation**

Random allocation sequence was performed by our methodologist colleague through Sealed Envelope website. Eligibility assessment of the patients and their allocation was conducted by the emergency resident under the supervision of the main person responsible for the project.

**Blinding**

In this study, the patients and the one who was responsible for measuring the desired outcomes in different groups were both blind to group allocation of patients. 

***Intervention***

An emergency medicine specialist randomly divided the patients into 2 groups. In the first group, the patients were treated with the standard protocol of 0.1 mg/Kg of intravenous morphine sulfate, 30 mg of intravenous ketorolac, and 100 ml intravenous normal saline, as placebo, within 15 minutes. In the second group, along with the standard protocol, 15 mg/Kg of intravenous magnesium sulfate 50% in 100 ml normal saline was additionally infused within 15 minutes (9).

**Outcomes**

Severity of patients’ pain was measured by VAS (7) at baseline as well as 30 and 60 minutes after infusion. The patients’ vital signs (blood pressure, pulse rate, respiratory rate, and arterial oxygen saturation) as well as possible side effects such as nausea, vomiting, dizziness, itching and drowsiness were recorded for all patients at minutes 30 and 60. In the case of VAS greater than 5 after 30 minute, additional dose of intravenous morphine sulfate was infused in both groups. The amount of the morphine administered to the patients until pain relief was recorded as well. Data was recorded by the assistant emergency medicine specialist, who was not aware of the groupings. Moreover, to check for the vital signs and side effects, up to 6 hours after the end of the study, the patients were followed up by checking their blood pressure, heart rate, respiratory rate, and deep tendinous reflexes. 

Clinically significant pain relief was considered as ≥ 3 score decrease of pain severity on VAS. 

**Statistical analysis**

Based on the mean pain scores, in magnesium sulfate and normal saline groups (2.1 ± 1.8 and 1.2 ± 2.9, respectively) (10), alpha error of 5%, and the power of 90%, 50 patients in each group were considered as the sample size. 

The study analysis approach was that of intention to treat. Data were analyzed using STATA 13 software through *t*-test and repeated measure ANOVA. P value less than 0.05 was set as the significance level. 

## Results

***Baseline characteristics***

**Figure 1:**
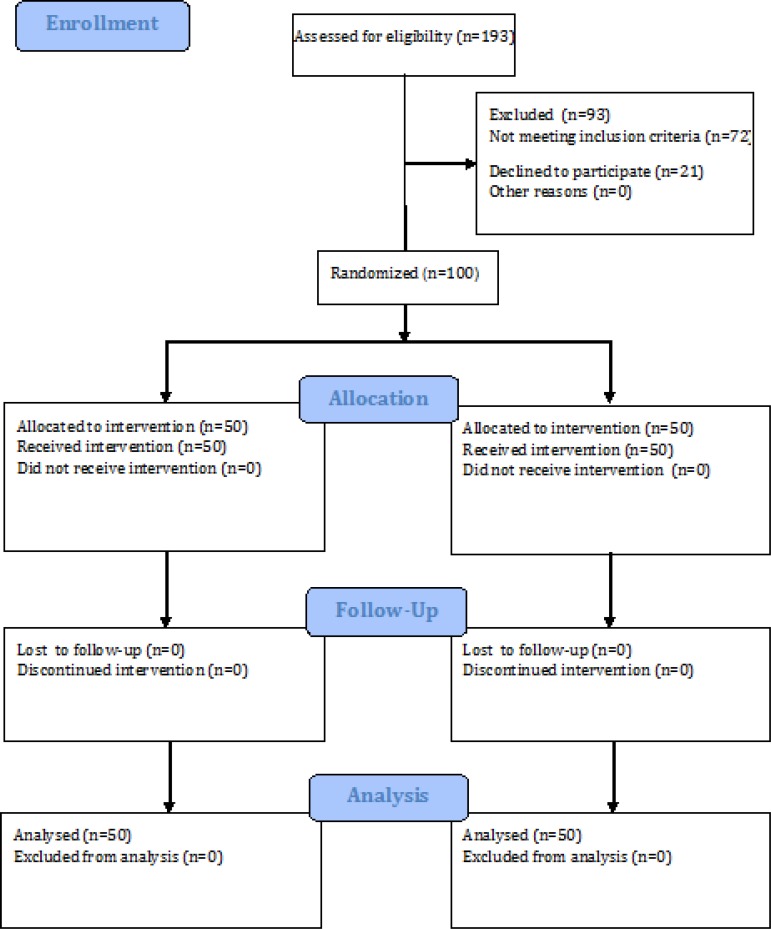
The flowchart of study.

100 patients (50 patients per group) were randomly allocated to two treatment groups. Patients’ flowchart is shown in figure 1. The baseline data for the two groups are shown in table 1. There was no significant difference between the two groups regarding baseline characteristics. Although a significant difference was found in body temperature of the patients in the two groups, such a difference is clinically negligible.

***Outcomes***

**Figure 2:**
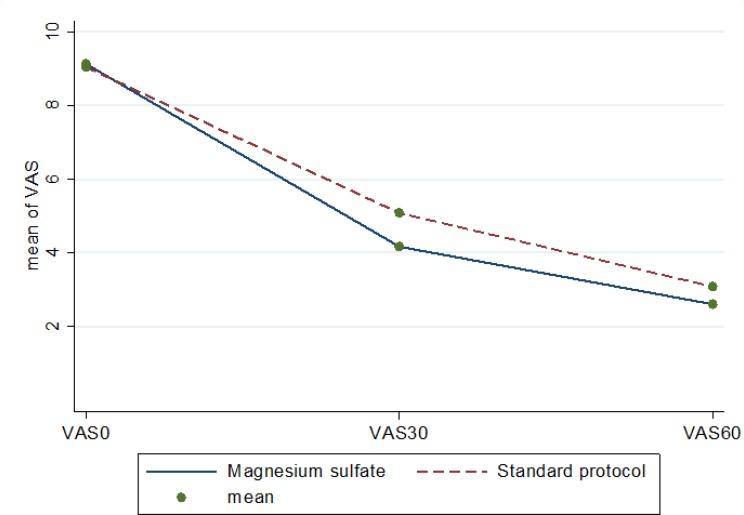
Mean pain severity (7) at various times in the two groups. VAS: visual analouge scale.

**Figure 3:**
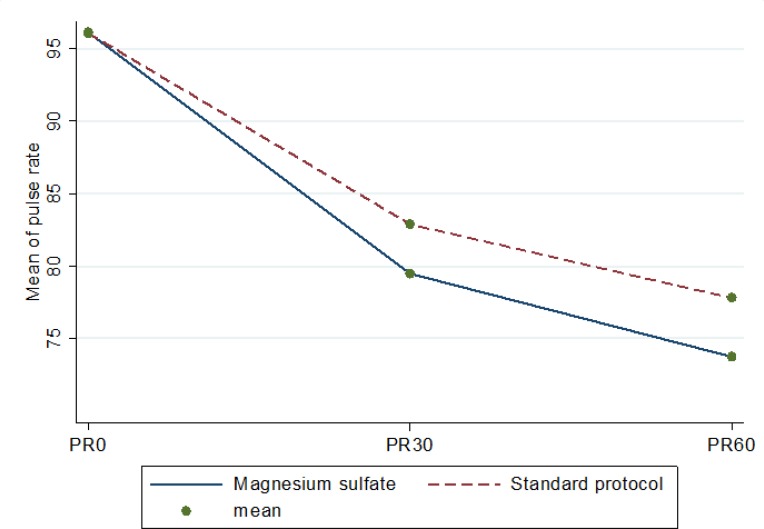
Mean pulse rate (20) at various times in the two groups. PR: pulse rate.

In table 2, the desired outcome of this study in the baseline, 30 and 60 minutes after intervention are compared between the two groups. Repeated measure ANOVA test showed no significant difference for systolic blood pressure (p = 0.677), diastolic blood pressure (p = 0.628), respiratory rate (p = 0.172), oxygen saturation (p = 0.933), and body temperature (p = 0.071) at various times between the two groups. The mean pain severity on VAS, however, was significantly different in the groups after intervention (p = 0.001; figure 2 and table 2). In addition, mean pulse rate in the magnesium sulfate group (p = 0.001) was significantly lower than that of standard protocol group (figure 3).

No significant differences in terms of nausea, vomiting, itching and drowsiness were, observed between the two groups. Likewise, no cases of dizziness were reported in the groups.

The average additional morphine received, in the control group was significantly higher than that of the magnesium sulfate group (1.56 vs 0.96 mg; p = 0.043).

**Table 1:** Comparison of baseline characteristics between intervention (magnesium sulfate) and control (standard protocol) groups

**Variables**	**Study groups**		**p value**
	**Intervention**	**Control**	
**Sex (male)**	30 (60%)	29 (58%)	0.839
**Age (year)**	33.64±8.61	35.16±8.97	0.389
**Weight (Kg)**	73.72±6.25	74.84±8.21	0.444
**Height (20)**	172.24±5.47	171.36±6.40	0.462
**Systolic BP (mmHg)**	137.56±7.93	137.36±10.61	0.915
**Diastolic BP(mmHg)**	81.88±6.83	82.16±6.68	0.836
**Pain severity (7)**	9.12±0.77	9.04±0.83	0.619
**Pulse rate (1/minute)**	96.12±4.69	96.08±3.42	0.961
**Respiratory rate(1/minute)**	17.24±1.34	17.04±1.41	0.470
**O**_2_ ** Saturation (%)**	93.88±1.79	93.72±1.77	0.654
**Temperature (Celsius)**	37.14±0.30	37.01±0.16	0.010
**Morphine dose (mg)**	6.88±0.71	6.96±0.96	0.639

Data were presented as mean ± standard deviation or number and percentage. BP: blood pressure, VAS: visual analogue scale.

**Table 2:** Mean hemodynamic measures and pain severity at different times for intervention (magnesium sulfate) and control (standard protocol) groups

**Variables**	**Study groups**				**p value**
	**Intervention**	**Control**			
**Systolic blood pressure (mmHg)**					
Baseline	136.56 (7.98)	137.36 (10.61)			0.677
30 minute	129.28 (8.32)	131.0 (8.10)			
60 minute	125.2 (6.40)	125.56 (9.42)			
**Diastolic blood pressure (mmHg)**					
Baseline	81.88 (6.83)	82.16 (6.68)			0.628
30 minute	76.88 (7.23)	78.08 (7.66)			
60 minute	73.80 (6.67)	74.28 (7.76)			
**Pain severity (7)**					
Baseline	9.12 (0.77)	9.04 (0.83)			0.001
30 minute	4.16 (0.88)	5.08 (1.50)			
60 minute	2.60 (0.49)	3.08 (0.56)			
**Pulse rate (1/minute)**					
Baseline	96.12 (4.69)			96.08 (3.42)	0.001
30 minute	79.48 (3.48)			82.88 (4.34)	
60 minute	73.72 (2.79)			77.80 (5.97)	
**Respiratory rate (1/minute)**					
Baseline	17.24 (1.34)			17.04 (1.41)	0.172
30 minute	14.52 (1.11)			15.32 (1.60)	
60 minute	13.56 (1.34)			14.04 (1.77)	
**O**_2_ ** Saturation (%)**					
Baseline	93.88 (1.79)		93.72 (1.77)		0.933
30 minute	94.28 (1.67)		94.20 (1.64)		
60 minute	94.12 (1.59)		94.28 (1.57)		
**Temperature (Celsius)**					
Baseline	37.14 (0.30)		37.01 (0.16)		0.071
30 minute	37.06 (0.20)		36.99 (0.13)		
60 minute	37.09 (0.28)		37.09 (0.28)		

## Discussion

The findings of present study suggest that adding magnesium sulfate to standard protocol of renal colic management could be effective in reducing the patients’ pain and need for additional dose of morphine sulfate without disturbing hemodynamic measures. However, the amounts of these effects are not clinically significant.

Magnesium plays key roles in several physiological processes. It has been stated to potentiate lidocaine (7), induce analgesia during spinal anesthesia (11, 12), improve morphine analgesia (13, 14), and reduce consumption of postoperative morphine (15, 16). Numerous studies with diverse doses, routes, and methods of administration of magnesium have been conducted with contradictory results (17-19).

Examining the impact of low-dose magnesium sulfate infusions on pain after laparoscopic cholecystectomy surgery, Kocman et al. (20) found that magnesium sulfate significantly reduces post-operative pain. 

In 2006, Safdar et al. (21) study showed that a combination of morphine and ketorolac relief pain better to either drug alone.

Rezae et al. (2014) examined the effect of magnesium sulfate infusion on pain relief after cesarean section and declared that infusion of 50 mg/kg magnesium sulfate reduces the pain and diminishes the need for other pain medications as well (10). 

Studying the bronchodilating effect of intravenous magnesium sulfate in bronchial asthma, Okayama et al. (22) also found that magnesium sulfate infusion can bring about rapid and significant dilation of the bronchi in both mild and severe asthma. Their study showed that magnesium sulfate relaxes the smooth muscles of the bronchial wall and dilates the ducts. 

In our study, adding magnesium sulfate to the standard treatment of patients with renal colic reduced the severity of pain and decreased the need for additional morphine. Considering that no side effects have been reported for using magnesium sulfate, as well as its easy application, this drug can be used as an adjunct drug in treatment of patients suffering from renal colic. Simultaneous use of magnesium sulfate with other drugs can also reduce their dosage and possible side effects. 

**Limitation**

The main limitation of this study was sample size. Due to small sample size and maybe low statistical power, it is recommended that further studies with larger sample sizes be conducted to examine any possible side effects for this drug and to confirm or reject the findings of this study.

## Conclusion

The results of this study indicated that magnesium sulfate can be used as an adjunct drug in treatment of patients suffering from renal colic. It can reduce the pain and diminish the need for additional doses of morphine sulfate without disturbing hemodynamic measures. However, the amounts of these effects are not clinically significant.
